# Attenuation of vaccinia virus by the expression of human Flt3 ligand

**DOI:** 10.1186/1743-422X-7-109

**Published:** 2010-05-26

**Authors:** Kamila Zurkova, Petr Hainz, Jitka Krystofova, Luda Kutinova, Miloslav Sanda, Sarka Nemeckova

**Affiliations:** 1 Institute of Hematology and Blood Transfusion, Department of Experimental Virology, U Nemocnice 1, CZ-128 20 Prague 2, Czech Republic; 2 Institute of Organic Chemistry and Biochemistry, Flemingovo nám. 2, CZ-160 00 Prague 6, Czech Republic

## Abstract

**Background:**

Vaccinia virus, one of the best known members of poxvirus family, has a wide host range both *in vivo *and *in vitro*. The expression of Flt3 ligand (FL) by recombinant vaccinia virus (rVACV) highly influenced properties of the virus in dependence on the level of expression.

**Results:**

High production of FL driven by the strong synthetic promoter decreased the growth of rVACV in macrophage cell line J774.G8 *in vitro *as well as its multiplication *in vivo *when inoculated in mice. The inhibition of replication *in vivo *was mirrored in low levels of antibodies against vaccinia virus (anti-VACV) which nearly approached to the negative serum level in non-infected mice. Strong FL expression changed not only the host range of the recombinant but also the basic protein contents of virions. The major proteins - H3L and D8L - which are responsible for the virus binding to the cells, and 28 K protein that serves as a virulence factor, were changed in the membrane portion of P13-E/L-FL viral particles. The core virion fraction contained multiple larger, uncleaved proteins and a higher amount of cellular proteins compared to the control virus. The overexpression of FL also resulted in its incorporation into the viral core of P13-E/L-FL IMV particles. In contrary to the equimolar ratio of glycosylated and nonglycosylated FL forms found in cells transfected with the expression plasmid, the recombinant virus incorporated mainly the smaller, nonglycosylated FL.

**Conclusions:**

It has been shown that the overexpression of the Flt3L gene in VACV results in the attenuation of the virus *in vivo*.

## Background

Vaccinia virus (VACV) is the best-studied member of the Orthopoxvirus genus of the poxvirus family. It has a wide host range and is able to infect cells of many different origins. VACV has played important roles in medicine and biomedical research. As VACV highly stimulates both the innate and adaptive arms of the immune system, it was used as the vaccine for eradication of smallpox and recently, the virus has been used as a live recombinant vaccine for the induction of protective immune response against many pathogens in experimental animals. VACV genome consists of a of 190 kbp dsDNA encoding over 200 proteins. The non-essential genes are used for the insertion of our gene of interest [1]. The resultant recombinant virus (rVACV) usually expresses foreign genes without remarkable impact on viral infectivity. Recombinant proteins are correctly posttranslationally modified, properly localized or secreted from infected cells.

Flt3 ligand (FL) is a hematopoietic growth factor that plays an important role in the life cycle of several blood cells. It is produced by bone marrow stromal cells, T cells and endothelial cells and by a number of organs including spleen, ovary, testis, intestine and kidney. FL alone induces differentiation of macrophages in CD34+ cell culture and stimulates increase in dendritic cell numbers [2-8]. When FL is administered to mice, hematopoietic stem cells and progenitors in the bone marrow and spleen are expanded and mobilized into the peripheral blood. FL increases beta-1-integrins or P-selectin expression and downregulates VCAM-1 on peripheral blood and folicular cells [9-13]. Moreover, the ligand acts in synergy with other cytokines, including stem cell factor (SCF), granulocyte-macrophage colony-stimulating factor (GM-CSF) and interleukins 3, 6, 7, 11 and 15. Stimulation by FL leads to proliferation, differentiation, maintenance and long-term reconstitution of primitive hematopoietic cells (both lymphoid and myeloid progenitors) [5,14-16]. FL dramatically enhances the production of antibodies to soluble antigens *in vivo *[17]. Systemic inoculation enhances the production of IFN-γ, IL-12, GM-CSF and IL-5 which results in increase of cytotoxic T lymphocytes, natural killer cells and dendritic cells in blood [18-20].

Human FL shares high homology with mouse FL in themino acid sequence, mainly in the extracellular part of the molecule, and is able to activate mouse Flt3 receptor [21]. The human Flt3L gene encodes a 235-amino acid type I transmembrane protein consisting of four domains: 1) an N-terminal 26-residue signal peptide, 2) a 156-residue extracellular domain, 3) a 23-amino acids transmembrane domain, and 4) a 30-residue cytoplasmic domain [4,15,22]. FL is expressed in membrane-bound and soluble forms. The cytokine is biologically active both in the transmembrane form and in the soluble form that is thought to be released into the circulation from the cell membrane by protease cleavage or is produced directly as the alternatively spliced soluble isoform [15,22-24]. The extracellular domain alone has been shown to be sufficient for bioactivity [23]. FL exists in both monomeric and homodimeric forms. Soluble FL can be a noncovalently linked oligomer and contains six cysteine residues in each molecule that apparently form intramolecular disulfides. The integrity of the FL dimer seems to be essential for bioactivity; moreover, the fusion of two soluble FL molecules can increase the activity of the ligand [25,26]. FL belongs to the family of short chain helical cytokines where the three-dimensional structures of five members, i.e. interleukin-4 (IL-4), IL-2, IL-5, GM-CSF and MCSF, have been solved [27]. The FL monomer has the most similar protein structure to IL-4 although the effects on blood cells are of different type [25,28].

The FL receptor, Flt3 (Fms-Like Tyrosine kinase 3), belongs to members of the class III receptor tyrosine kinase family of transmembrane glycoproteins and is structurally related to the c-kit (KIT), c-fms (FMS), and platelet-derived growth factor (PDGF) receptors. The receptor is expressed only in a limited number of tissues, including the human bone marrow, thymus, spleen, liver, and lymph nodes.

In this study, we examined the influence of FL production on the life cycle of recombinant virus. We constructed two recombinant vaccinia viruses of the Praha strain (clone P13) designed for the expression of the human gene encoding a soluble isoform of FL (sFL). The rVACVs were characterized and compared for their multiplication and virulence *in vitro *and *in vivo*, and for the ability to ensure the secretion of FL from infected cells.

We found out that the FL overexpression substantialy influenced the properties of rVACV. High production of FL resulted in decreased rVACV multiplication in macrophages and in mice. Biochemical and electron microscopic analysis of the recombinant virions revealed changes in the protein composition and incorporation of FL into the virion core. We have shown that the overexpression of the Flt3L gene in VACV results in attenuation of the virus *in vivo*.

## Results

### Virus multiplication and sFL production and localization in vitro

**Figure 1:**
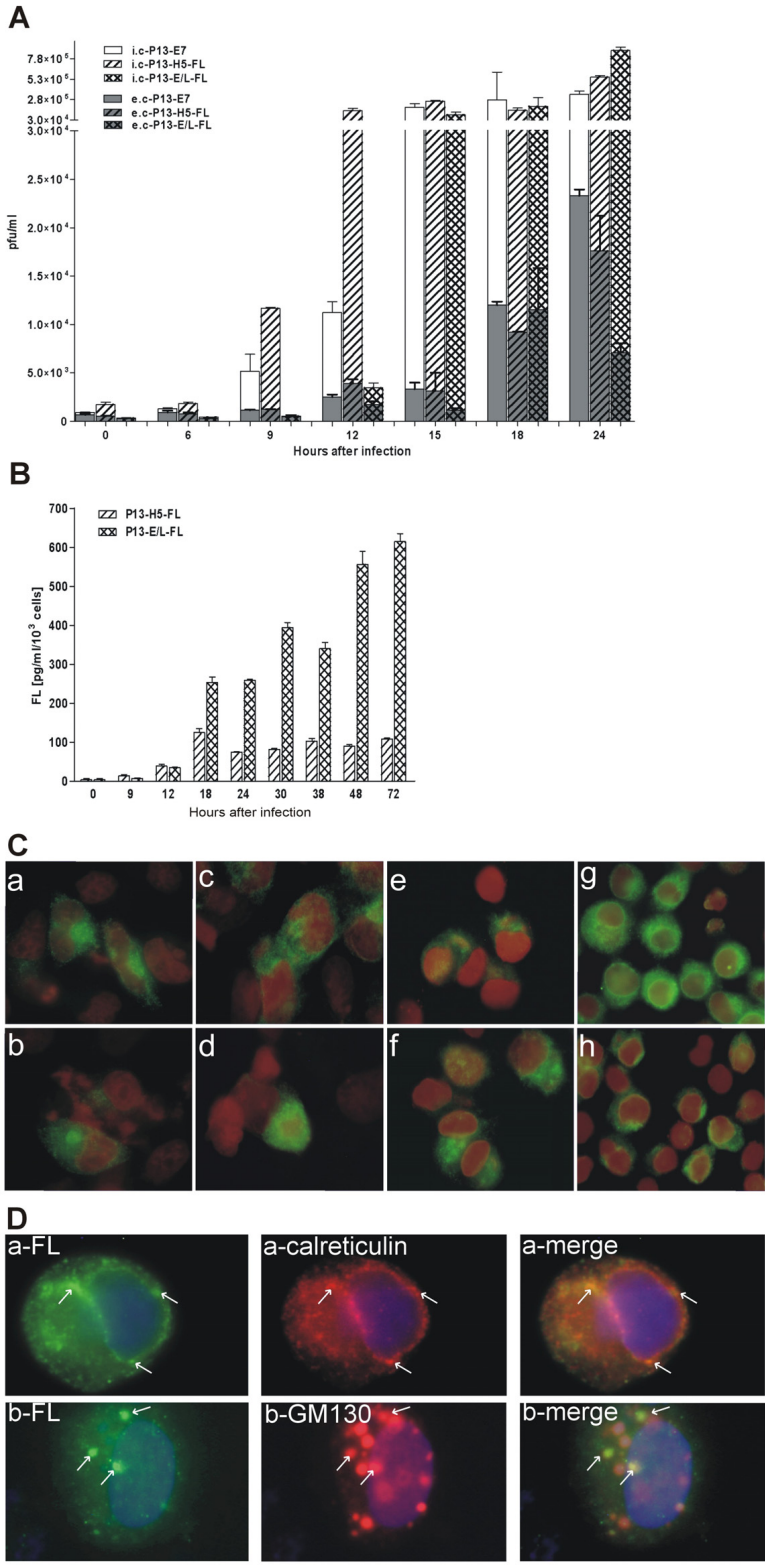
***In vitro *multiplication and sFL production by rVACV**. Confluent CV-1cell cultures were infected with purified virus at a MOI of2.5 at 37°C for 1 hour, washed with PBS and the fresh medium was added. The medium and cells were collected, frozen and thawed, and cell debris was removed. The titers of the infectious virus **(A) **were determined in the medium and in the cell lysate. Total FL production was determined by ELISA **(B)**. The columns in all graphs represent the mean ± s.d. The intracellular location of sFL **(C) **in 293T cells **(a, b) **48 h after transfection or in infected CV1 **(c, d) **and HeLa cells **(e, f) **9 h after infection or in J774.G8 cells **(g, h) **3 h after infection with P13-H5-FL **(c, e, g) **or P13-E/L-FL **(d, f, h) **as visualized by an immunofluorescent microscope at a magnification of 1000×. The colocalisation of sFL **(D) **with endoplasmic reticulum marker calreticulin or with cis-Golgi marker GM130 in HeLa cells 3 h after infection as visualized by an immunofluorescent microscope at a magnification of 1000×.

We prepared recombinant vaccinia viruses expressing the gene for a soluble isoform of human Flt3 ligand (FL). In the first instance, we determined FL production and virus multiplication *in vitro*. The monolayers of CV1 cells were infected with recombinant VACV at a MOI of 2.5 and cells were cultured in the fresh medium. The medium and cells were harvested at the indicated intervals (Fig 1). The cells were frozen and thawed and the centrifuged supernatant was removed from cell debris. Both, media containing extracellular (e. c) virus and supernatants containing intracellular (i. c) virus were used for determination of infectious virus or production of FL. Replication of recombinant viruses was determined by plaque assay. The concentration of FL was measured by ELISA test.

The overall multiplication of FL-expressing recombinants and control virus in CV1 cells was similar (Fig. 1A); however, the newly formed infectious particles of P13-E/L-FL were retained in infected cells and released to the medium at later intervals after infection as compared with P13-H5-FL or control virus P13-E7. Similar rates of viral multiplication in CV1 cells were also found for double recombinant viruses expressing β-galactosidase gene of *E.coli *(data not shown). The production of intracellular and secreted FL was followed up to 72 h.p.i. (Fig. 1B). The peak of FL production driven by the H5 promoter was at 18 h.p.i., whereas the production from the E/L promoter increased gradually and high amount of FL was produced up to very late intervals.

To determine the sites of production, intracellular storage and transport of sFL protein in infected cells, the sFL was detected by immunofluorescent staining. Confluent cultures of CV1 and HeLa cells were infected with P13-H5-FL or P13-E/L-FL or control virus at a MOI of 2 for 30 minutes, cultured in fresh medium and stained at several time intervals. To compare the location of sFL protein expressed by rVACV and produced in the absence of virus infection, 293T cells (6 × 10^4^) were transfected with the expression plasmid pBSC-FL (2.5 μg) where the expression of the sFL gene was controlled by the strong cytomegalovirus promoter. Transfected cells were processed for fluorescent microscopy after 48 hours of cultivation. The localization of sFL in the transfected 293T cells (Fig. 1C-a, b) had the same fluorescent pattern as in the virus infected cells (Fig. 1C-c, d, e, f). The course of the sFL fluorescent signal in infected cells correlated in time with strength of expression directed by the H5 or E/L promoter (not shown). The H5 promoter was stronger in early phase of infection and the fluorescent signal was already visible at 3 hours after infection while the expression driven by the E/L promoter was hardly observable. The signal of the sFL expression controlled under the E/L promoter was stronger at later time intervals (followed up to 12 h.p.i.) when the signal from P13-H5-FL infected cells did no more rise (not shown). When we stained the specific markers for cellular organels, calreticulin and GM130, we observed the sFL protein localized mainly in the endoplasmic reticulum (Fig. 1D-a). The colocalisation of sFL with the cis-Golgi marker GM130 (Fig. 1D-b) was not so obvious in infected cells and decreased in time due to a massive transition of GM130 to viral factories. The considerable portion of sFL seemed to be transported in the vesicles toward the cell surface.

### Multiplication of P13-E/L-FL in vivo as well as in macrophage cell line J774.G8 is impaired

To determine the influence of FL expression on viral multiplication *in vivo*, we infected mice intraperitoneally with 1 × 10^6 ^pfu of rVACV. The ovaries and blood serum were collected at 24-hours intervals. The virus in ovaries was determined by plaque assay of tissue homogenate or as viral DNA by quantitative PCR. The level of FL was measured by ELISA in diluted sera.

**Figure 2:**
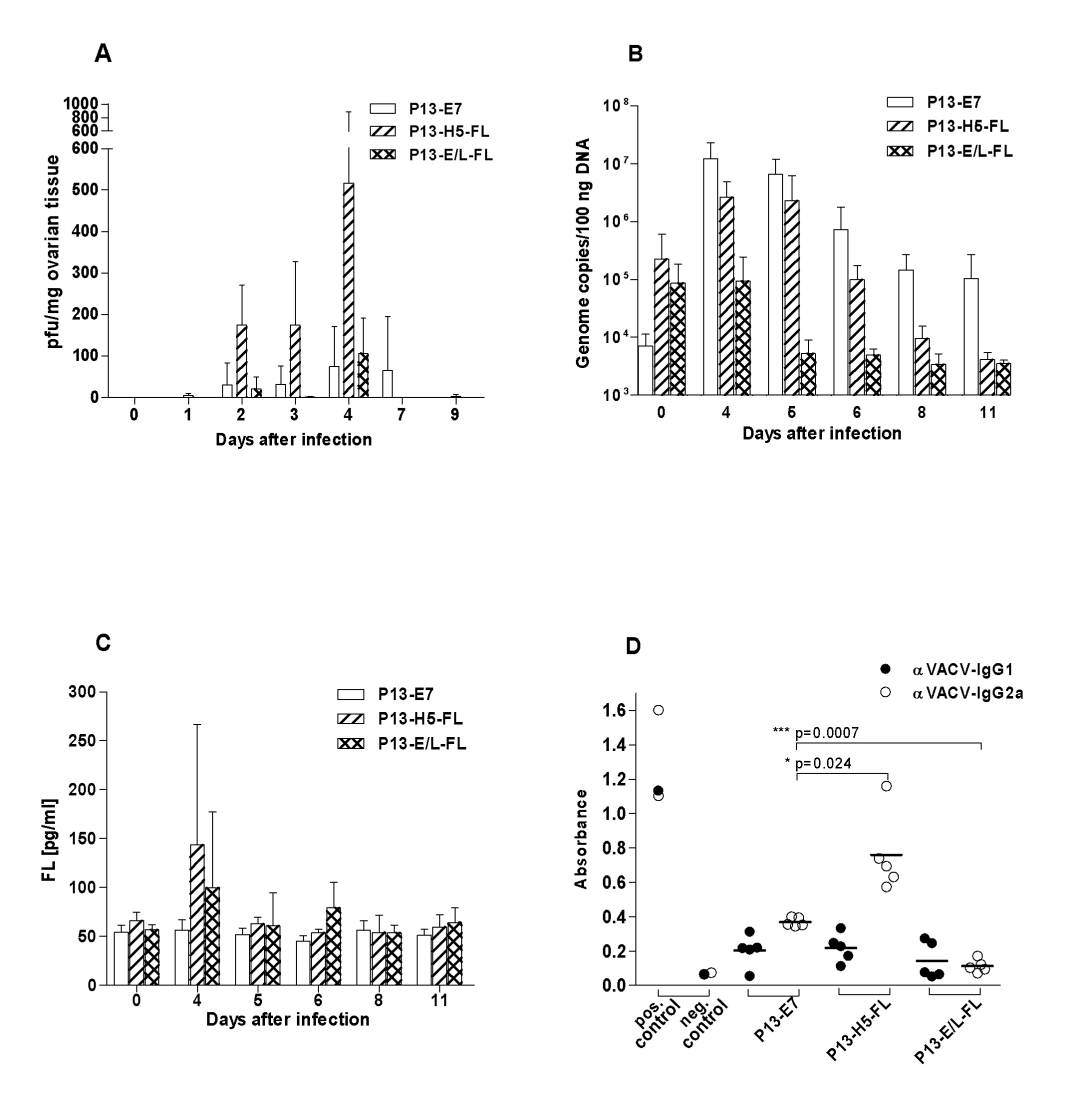
***In vivo *multiplication, sFL production and antibody response to rVACV**. Groups (n = 4) of C57Bl/6 female mice were inoculated i.p. with 1 × 10^6 ^pfu of P13-E/L-FL, P13-H5-FL or P13-E7. The ovaries and sera were collected at various intervals after infection. Replication of rVACV in the ovaries was determined by plaque assay **(A) **or by Q-PCR **(B)**. The *in vivo *production of FL was measured in mouse sera by ELISA **(C)**. To determine the immunogenicity of recombinants, groups of mice (n = 5) were i.p. inoculated with 1 × 10^6 ^pfu of rVACV. Five weeks later, the animals were anesthesized and the serum was collected. The levels of VACV specific IgG1 or IgG2a antibodies were quantified by ELISA **(D)**. The data were analyzed by the two-way ANOVA.

The amount of virus (Fig. 2A) and viral DNA (Fig. 2B) in the ovaries was increasing from the time of inoculation to day 4 and then dropped. The comparison of multiplication between different viruses showed that the expression of the FL gene did not affect multiplication of P13-H5-FL virus, whereas the growth of P13-E/L-FL virus was impaired in mouse ovaries, contrary to the situation in CV1 cells *in vitro*. Similarly, serum FL was higher in animals infected with P13-H5-FL virus in comparison with P13-E/L-FL (Fig. 2C). The highest amount of FL in the serum was found on day 4 after infection in accordance with the strongest virus replication in mouse ovaries. The decreased serum FL levels reflected the impaired replication of the recombinant virus expressing FL under the control of the E/L promoter as detected in the mouse ovaries.

In order to determine the impact of the attenuation of *in vivo *multiplication of P13-E/L-FL on its ability to induce immune response, we measured the amount of IgG1 and IgG2a specific for VACV by ELISA (Fig. 2D) in the mouse sera 5 weeks after virus inoculation. We observed that the multiplication of viruses correlated with the level of anti-VACV antibodies. P13-E/L-FL induced no detectable IgG1 or IgG2a antibodies against the vaccinia antigen, whereas P13-H5-FL and P13-E7 elicited anti-VACV specific antibodies predominantly of IgG2a isotype as expected for the immune response against vaccinia virus infection.

**Figure 3:**
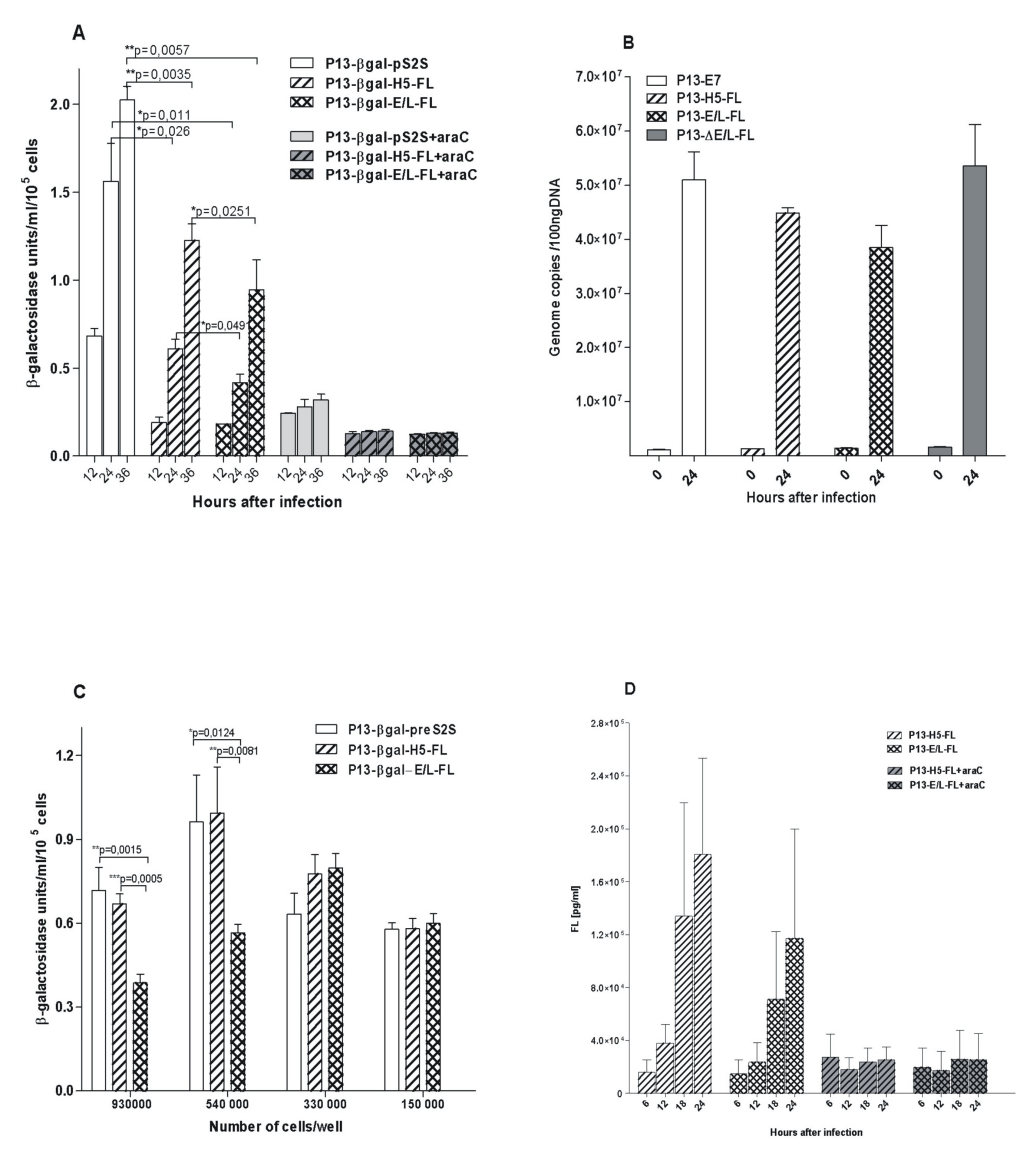
**Multiplication of rVACV and sFL production in macrophage cell line J774.G8**. Culture of J774.G8 cells was infected with the purified virus at MOI of 2.5 **(A, C, D) **or at MOI of 0.1 **(B) **at 37°C for 1 hour, washed with PBS and the fresh medium with or without cytosine arabinoside (40 μg/ml) was added. Multiplication of virus was determined as beta-galactosidase activity **(A, C) **or by Q-PCR **(B) **at indicated intervals (A, B) or 24 hours after infection (C). Total FL production was determined by ELISA **(D)**.

To find an *in vitro *model for the study of *in vivo *inhibition of P13-E/L-FL replication, we compared the ability of recombinants expressing sFL to multiplicate in cell lines of various origin such as mouse J774.G8 macrophages, 32D cells, human HeLa cells or MOLM-9 leukemic cells. We infected cells at a MOI of 1 with double recombinant viruses expressing FL or HPV16 E7, together with β-galactosidase serving as a reporter gene for relative multiplication of viruses. The infected cells were incubated with or without cytosine arabinoside (40 μg/ml) to block viral DNA synthesis and consequently to prevent late gene expression [29]. The β-galactosidase activity was monitored up to 36 hours post infection. We found out that FL expression did not affect rVACV multiplication in HeLa cells, as the β-galactosidase activity was similar for all viruses at every interval (not shown). Mouse and human cell lines of hematopoietic origin, 32D and MOLM-9, were completely non-permissive for any of the rVACV tested (not shown). However, a similar situation as *in vivo *has been observed during infection of confluent culture of macrophage cell line J774.G8. In comparison with the other double recombinants, multiplication of P13-βgal -E/L-FL virus in J774.G8 was reduced as determined by β-galactosidase assay (Fig. 3A), quantitative PCR of viral DNA (Fig. 3B) and titration of viral progeny produced by the single recombinants (not shown). The inhibition of FL-expressing viruses was slightly visible also in samples with added ara-C where only the early production of β-galactosidase occurred. Deletion of the FL gene in P13-ΔE/L-FL resulted in restoration of multiplication of the reversion mutant (Fig. 3B). The relative multiplication of P13-βgal-E/L-FL virus, measured as the β-galactosidase production, depends on macrophage culture conditions. In quickly growing cells (about 40% confluence), we observed no inhibition of P13-βgal-E/L-FL (Fig. 3C), whereas the growth of P13-βgal-E/L-FL virus in wells containing more than 5 × 10^5 ^J774.G8 cells was significantly restricted comparing with P13-βgal-H5-FL or P13-E7 virus.

To determine the effect of the attenuation of P13-E/L-FL in macrophages J774.G8 on transgene expression, we measured sFL production by infected cells. Confluent culture of J774.G8 cells was infected at a MOI of 2.5 and secretion of sFL into the medium as well as the amount of intracellular FL was measured by ELISA (Fig. 3D). We noticed that FL production directed by the strong E/L promoter was lower but nearly comparable with that under the control of the H5 promoter. This result was in accordance with the reduced multiplication of the P13-E/L-FL recombinant in macrophage culture. The amount of produced FL was not influenced in early phase of infection as showed bars of samples with added ara-C, which support the idea that the inhibition of virus expressing sFL under E/L promotor occures in replicative and postreplicative phases of infection. The localization of FL in infected macrophages (Fig. 1C-g, h) determined by immunofluorescence was identical to that observed in other tested cell types. The fluorescence signal was visible as early as 90 minutes after infection and persisted during the observed time interval. At 3 hours after infection, the signal was weaker in the macrophages infected with P13-E/L-FL compared to P13-H5-FL.

### The core of intracellular mature virions (IMV) harbours sFL

**Figure 4:**
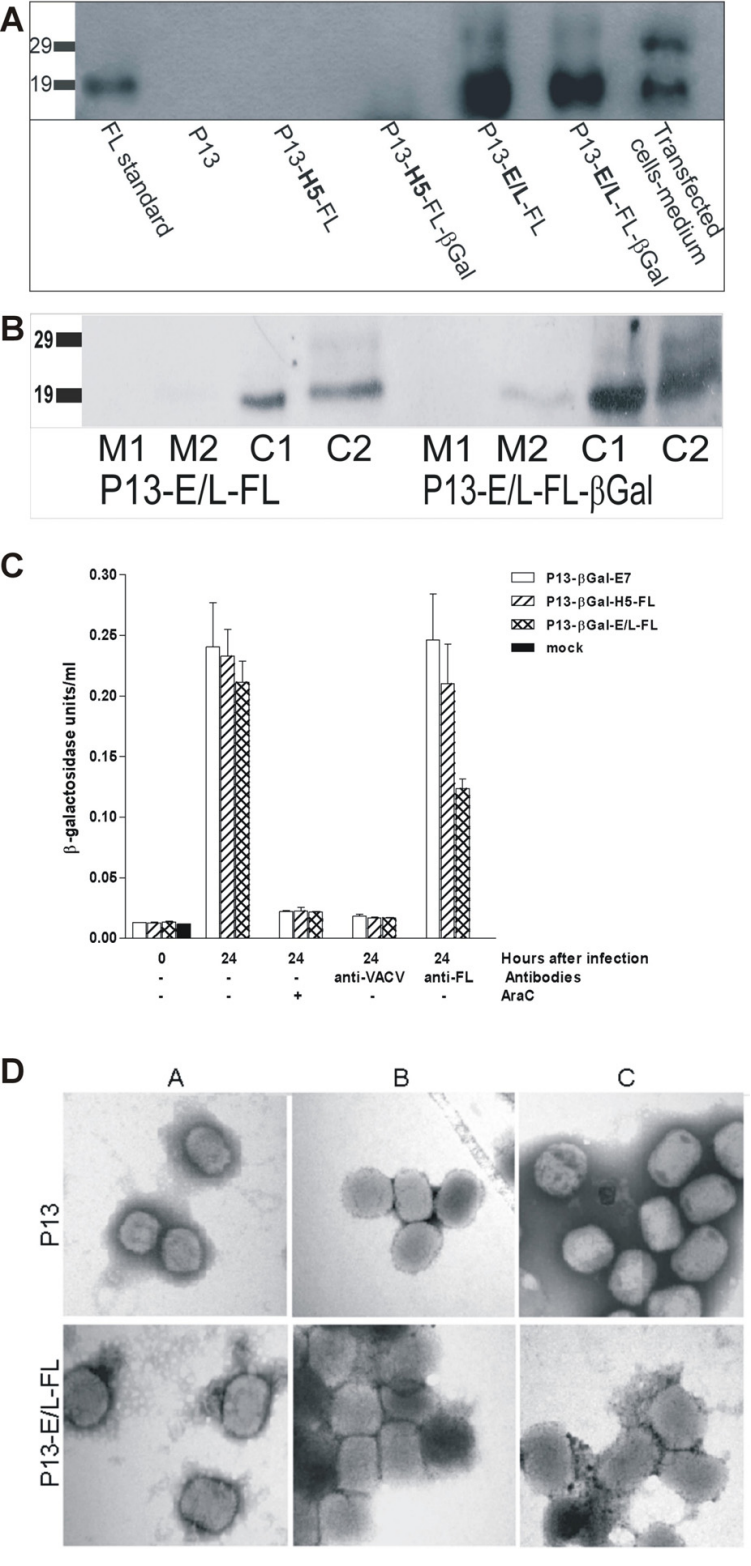
**Incorporation of sFL into viral particles**. Aliquots of the indicated sucrose purified virions **(A) **or their solubilized and separated fractions **(B) **were analyzed by western blot using the FL specific BAF308 monoclonal antibody. The sample prepared from the culture medium of 293T cells transfected with pBSC-FL expression plasmid served as a positive control. To neutralize virus infectivity, the saccharose purified particles were incubated at 37°C for 1 hour with PBS, anti-FL antibody or anti-VACV serum. The first aliquot of samples was used for infection of cell cultures **(C)**. Cytosine arabinoside (40 μg/ml) was added for inhibition of viral replication as the positive control. The beta-galactosidase assay was performed after 24 hours of cultivation. The second aliquot of samples was applied to a formvar membrane coated copper grid **(D)**. Negatively stained particles were examined under an electron-microscope at a magnification of 50 000×.

In the next step, we analyzed by western blots the composition of purified intracellular mature virions (IMV) of FL-expressing viruses, P13-H5-FL, P13-E/L-FL and double recombinants carrying both the sFL also β-galactosidase genes and of P13 parental virus (Fig. 4A) using FL specific antibody. We found out that high sFL expression driven by the E/L promoter resulted in the incorporation of sFL in virus particles, whereas sFL incorporation was not found if the expression was regulated by the H5 promoter. The sFL band associated with virion cores had the same size of 19 kDa as found for the low-glycosylated sFL produced in infected cells or in cells transfected with the expression plasmid pBSC-FL. The 25 kDa glycosylated sFL produced in infected and transfected cells was not detected in purified virions.

To analyze the composition of intracellular mature virions (IMV), we partitioned virions into two membrane (M1, M2) and two core fractions (C1, C2) and analyzed them by western blot using FL specific antibody (Fig. 4B). The enrichment of the membrane and core fractions was confirmed by western blot using antibodies to p16 (A14, membrane protein), 4b (A10, core protein) and convalescent anti-VACV mouse serum (not shown). We found sFL (19 kDa) predominantly in the core fractions and not in the membrane fractions of P13-E/L-FL IMV or other tested viruses.

To confirm the association of sFL with virions, we performed the neutralization assay. In this experiment, sucrose purified particles of the double recombinants carrying the β-Gal gene together with FL or a control gene were incubated with polyclonal antibody against FL or with anti-VACV rabbit or mouse serum or non-immune serum. The neutralization of virus infectivity was measured as the ability to produce beta-galactosidase in CV1 cells (Fig. 4C). The control virus P13-βgal-E7 as well as P13-βgal-H5-FL and P13-βgal-E/L-FL viruses were neutralized by incubation with anti-VACV mouse or rabbit serum. Neither P13-βgal-E7 nor P13-βgal-H5-FL was neutralized by anti-FL antibodies. The infectivity of P13-βgal-E/L-FL virus was lowered by anti-FL antibodies, but not to such an extent as as observed using the positive anti-VACV serum.

We speculated that a portion of sFL was displayed on the surface of IMV, and we used the same antibodies or sera to confirm the results of the neutralization experiment by direct observation of virions. Purified virions of P13-E/L-FL or P13 viruses were incubated with sFL specific polyclonal antibody or rabbit and mouse anti-VACV serum or non-immune serum. The virus particles were negatively stained and examined under an electron microscope (Fig. 4D). The anti-VACV sera were able to aggregate viral particles of both viruses. The anti-FL antibody mediated aggregation of P13-E/L-FL particles but not of the parental P13 virus.

### Integration of sFL in virions is associated with aberrant protein composition of viral particles

**Figure 5:**
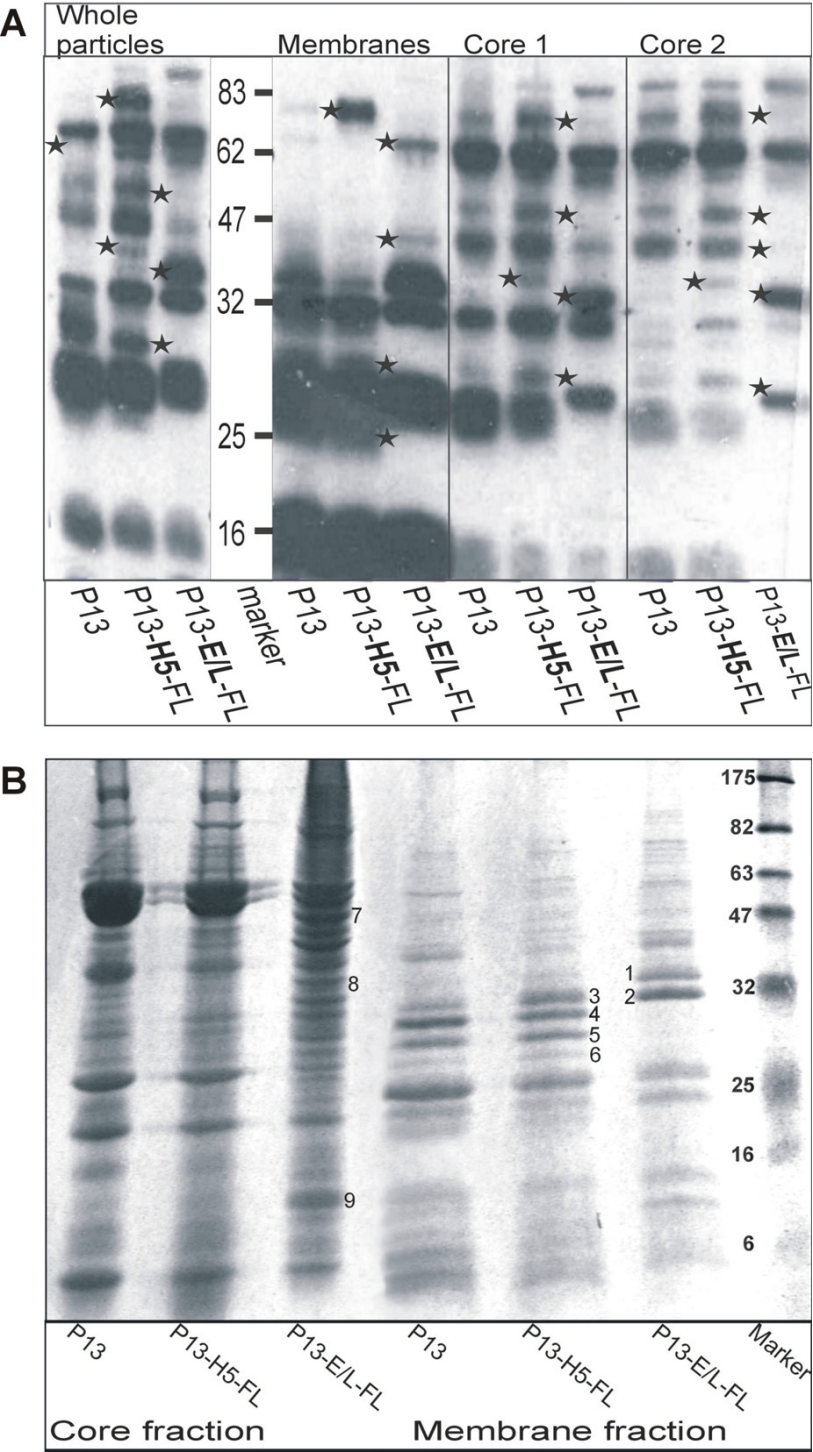
**Analysis of virion protein composition**. Purified P13, P13-H5-FL or P13-E/L-FL viruses were fractionated into enriched membrane and core fractions. Aliquots were separated by 12% or 8-15% gradient SDS-PAGE. The gel was processed by western blotting and stained with anti-VACV mouse serum **(A) **or stained with Coomassie blue **(B)**. The proteins of differing bands were identified by MALDI.

As the P13-E/L-FL virions were distinct in structure from P13-H5-FL virus particles, we looked for other differences in the protein composition. The sucrose purified particles were fractionated in similar way as used for FL protein detection above and afterwards analyzed by Western blot using anti-VACV mouse serum (Fig. 5A). The analysis revealed additional and missing distinct immunodominant VACV protein bands (marked with asterisks) in the membrane as well as in the core fractions of P13-E/L-FL IMV in comparison with P13-H5-FL or P13 virus. In order to uncover the differing proteins, SDS-PAGE gels were stained with Coomassie blue (Fig. 5B) and bands were cut out from the gel and identified by mass spectrometry (Table 1). The major membrane protein p35 (gene H3L, band 1) of P13-E/L-FL virus was shifted in comparison with two other viruses (band 4). Band 1 co-migrated with annexin 5. All three viruses contained the membrane protein p32 (gene D8L) detected in band 2 or 3; however, the membranes of P13 and P13-H5-FLviruses comprised an additional form of p32 detected as band 5. The P28K protein (gene L4R, band 6) was absent in the enriched membrane fraction of P13-E/L-FL, whereas P13 and P13-H5-FL contained a faint band of this major core protein. This band was also less abundant in the core fraction of P13-E/L-FL in comparison with other two viruses. Furthermore the core fraction of P13-E/L-FL virus harbored an additional hypothetical 10 kDa viral protein (band 9) and host proteins tubulin β chain (band 7) and glyceraldehyde-3-phosphate dehydrogenase (GAPDH) (band 8).

**Table 1:** Virion proteins identified in membrane- and core- enriched fractions detected by SDS-PAGE and mass spectrometry analysis.

# band	ORF	Protein function (Reference)	# of identified peptides
1	H3L	IMV major membrane protein p35 [50] + annexin 5	12

2	D8L	IMV membrane protein p32 [54]	7

3	D8L	IMV membrane protein p32	7

4	H3L	IMV major membrane protein p35	13

5	D8L	IMV membrane protein p32	8

6	L4R	Major core protein 28 k [73]	8

7	Cellular	Tubulin β chain	4

8	Cellular	Glyceraldehyde-3-phosphate dehydrogenase (GAPDH), EC1.2.1.1.	8

9	Putative AORFL	Hypothetical 10.8 kDa [74]	3

## Discussion

Several studies have shown that FL protein can inhibit tumor growth *in vivo *and acts as a vaccine adjuvant. Instead of direct FL protein administration, the production of FL by a viral vector might result in prolonged FL presence in the body and in improved therapeutic activity [30].

We constructed two recombinant VACVs derived from the Praha vaccine strain whose expression of FL was controlled by the natural early H5 promoter which is important for early antigen presentation, or by the synthetic E/L promoter with activity at both early and late times during VACV infection [31,32]. The original goal of our project was to use FL-expressing viruses for stimulation of antigen presenting cell activity and adaptive immunity in tumor bearing animals. Indeed, we have shown that immunization with VACV co-expressing sFL with the E7 protein of HPV16 as a tumor antigen inhibited the formation and growth of TC1 tumors in mice [33]. In that study we observed that despite high expression of FL controlled by the synthetic E/L promoter *in vitro*, the double recombinant P13-E/L-FL-SigE7LAMP induced only low levels of FL in the serum of inoculated mice. In an attempt to elucidate the nature of the inhibitory effect of FL overexpression controlled by the E/L promoter on the release of recombinant cytokine in mouse serum, we focused on a more detailed study of FL-producing recombinants.

The first step was to characterize the multiplication and FL production *in vitro*. We compared the replication of P13-H5-FL, P13-E/L-FL and control virus in CV1 cells by virus progeny titration. No distinct effect of recombinant protein expression on the replication was observed, apart from the moderately delayed release of P13-E/L-FL virus from infected cells. ELISA tests of media and cell lysates of infected cultures confirmed the generally accepted idea of the strength of natural H5 promoter of VACV which is active mainly in the early phase of infection and of the synthetic E/L promoter whose activity is increasing from the early to the late phase of infection.

Then we administered these viruses intraperitoneally to 6-weeks-old mice and measured the expression of FL *in vivo *for several days after inoculation. The animals inoculated with P13-E/L-FL did not have highly elevated serum levels of FL, similarly to the previous experiments done with double recombinant P13-E/L-FL-SigE7LAMP. Moreover, we found out, by titration and by quantitative PCR of viral DNA in mouse ovaries, that the P13-E/L-FL virus was not able to multiply *in vivo*. It was seemingly in contradiction with our previously published results where the expression of FL under the control of the E/L promoter did not affect the multiplication of the double recombinant P13-E/L-FL-SigE7LAMP in the mouse ovaries in comparison with the control virus and with the double recombinant P13-H5-FL-SigE7LAMP [33]. When comparing the double recombinant P13-E/L-FL-SigE7LAMP with the single recombinant P13-E/L-FL *in vitro*, we found the latter to produce higher levels of Flt3L in infected CV1 cells (not shown). Decrease in FL production by double recombinant *in vitro *could be ascribed to the inactivation of the F7L locus as a result of the insertion of the SigE7LAMP gene, which is known to down-regulate the expression of the gene inserted in the TK locus [34]. The overexpression of FL by the single recombinant was so high that it resulted not only in limited production of FL *in vivo *but even in its decreased multiplication in mice. The block of P13-E/L-FL multiplication was confirmed by the examination of the independently derived recombinants and by the deletion of the E/L-FL expression cassette followed by reversion to the wt phenotype. We checked also the DNA pattern of the genome of all analyzed viruses using SalI, HindIII, PstI, XhoI and KpnI endonucleases and found no obvious differences in the restriction patterns (not shown). It was evident that the inhibition of virus multiplication *in vivo *was caused by sFL overexpression under the control of the E/L promoter.

Attenuation of recombinant vaccinia vectors in consequence of the foreign gene insertion has been described for viruses expressing IL2 [35], IL12 [36] and IL15 [37]. The multiplication was inhibited *in vivo *and in lymphoid cell lines; however, the infected fibroblasts produced the control and cytokine-expressing viruses in equivalent titers.

The response to VACV infection has been studied in several species [38-40]. It has been illustrated recently in variola primate model that poxviruses productively infect large populations of circulating monocytes and macrophages in the lymph nodes, spleen and other tissues [41]. For our study, we selected as an *in vitro *target cell model the macrophage cell line J774.G8 which supports the growth of VACV. The multiplication of the P13-E/L-FL virus but not of the P13-H5-FL virus was restricted in confluent cultures of this cell line. Taken into consideration, this situation is likely to simulate the *in vivo *state where the macrophages are terminally differentiated cells that rarely divide. We also determined the level of FL expression in J774.G8 cells by P13-H5-FL or P13-E/L-FL. Despite the sFL expression driven by promotors of different strengths, we found the same FL secretion level by either virus. It could mean that the inhibition of virus multiplication is not mediated by high level of extrinsic FL. This fact has been supported by the failure to find any Flt3 (CD135) molecules on the membrane of J774.G8 cells and by experimental addition of extrinsic Flt3L to J774.G8 cells that had no effect on the multiplication of the control P13-preS2S-βgal or P13-E7 viruses at any step of infection.The inhibition of multiplication of P13-E/L-FL in macrophages might be reversible since we showed the dependence of β-galactosidase production by P13-βgal-E/L-FL on the growing activity of cell cultures.

To exclude the possibility that apoptosis is responsible for inefficient multiplication of P13-E/L-FL in macrophages, the presence of the apoptosis marker Annexin-V and the cleavage of PARP were determined. For these experiments, we prepared the double recombinants expressing both the FL gene and GFP protein and used them for following up the early apoptosis marker (Annexin-V) in infected cells during virus replication. There were not significant differences among viruses in the infectivity (GFP positive cells amount) or in Annexin-V binding (not shown). We also tested the cleavage of PARP protein (late apoptosis marker) in macrophages infected by single recombinant viruses. The cleavage was obvious in cells infected by parental virus or P13-H5-FL and slightly in P13-E/L-FL infected macrophages. As a control, we added ara-C to macrophages, which caused PARP cleavage in all infected and non-infected macrophages (not shown). We concluded that the attenuation of P13-E/L-FL was not due to the enhanced apoptosis in macrophages.

After challenging the assumption that the antiviral state of macrophages is mediated by high levels of Flt3L produced by recombinants during infection, we considered the possibility of changes in the elementary protein composition of the virion itself. The strong expression of FL driven by the synthetic E/L promoter led to its incorporation into a core-associated protein fraction. This was due to the strong FL expression in BSC40 or HeLa cells used for virus stock preparation. The endoplasmic reticulum and Golgi apparatus of P13-E/L-FL infected cells contained a huge amount of FL protein. The foreign gene product has been reported previously to be trapped in the virion due to protein-protein interactions during the virion assembly process [42-44]. Bereta et al. have indirectly shown that the rVV expressing CD40L gene contains biologically active CD40L protein in particles [45] However, there is some selectivity in the encapsidation process. There is evidence of the incorporation of recombinant protein into one or more virion compartments. Vaccinia virus expressing the bacterial CAT gene incorporated the enzyme into the virus particle [46]. The expression of the cytokine IL-12 led to the incorporation of about 0.01% of the total recombinant protein into an envelope fraction, HIV1 env (gp160-120) was tightly bound in protein-DNA complexes, and the enzyme beta-galactosidase was found exclusively in core-associated fraction [47]. Transport and sorting of viral proteins directly from the endoplasmic reticulum into the growing immature virions using non-COPII vesicles [48] could explain selective integration of the low-glycosylated FL into P13-E/L-FL virions. A similar selectivity for the integration of non-glycosylated form of a glycoprotein into the membrane of IMV particles was observed for A14 [49]. However, FL was not incorporated into a membrane fraction, but it was tightly bound to core, although it was also exposed on the surface of IMV to offer the epitopes for anti-FL antibodies as verified by neutralization assays and proven by electron microscopy. Moreover, the localization of the recombinant proteins may be facilitated by specific protein-protein interactions. Variation in the localization of recombinant protein has been reported in double recombinants expressing the protein of interest and β-galactosidase [47]. We also analysed the FL distribution also in double recombinant viruses P13-βgal-H5-FL and P13-βgal -E/L-FL and found no changes in comparison with the single recombinant viral particles.

In the present study, we have shown that the integration of sFL into the IMV virions was associated with an altered composition of the virions. We observed that P13-E/L-FL virions contained H3L of a higher molecular weight than P13 or P13-H5-FL (lanes 1 and 4). The H3 protein is an immunodominant component of IMV, binds to heparansulphate and is found in IMV in two isoforms, (1-324 aa) and (48-324 aa) [50]. Both the full length protein and N-terminally deleted form can be incorporated into the viral membrane [51] although the specific functions of the H3 isoforms are yet not known. As the H3 protein is nonessential for virus multiplication in cell monolayers [52], the integration of FL into the virion might modify the ratio of H3 isoforms. Similarly to H3, the second differently displayed virion component, the D8 protein, plays the role as the glycosaminoglycan (GAG) binding molecule [53]. It has been shown, that the D8 protein integrated into the viral particle can be cleaved by trypsin without any decrease of virus infectivity [54]. The D8 protein detected in band 5 could be a cleavage product of trypsin like protease. The unusual forms of both GAG binding structures resulting from high FL gene expression may influence the growth of virus in some cell types. Differences in these two membrane proteins in association with FL gene expression could imply that the presence of FL in the virus core also affects the composition of the IMV core. The pattern of core proteins showed multiple band changes. One dominant band contains the p28K protein encoded by L4R. This protein is a basic DNA binding protein and plays an essential role in virus replication. The protein fragment (33-251 aa) produced by Ala-Gly-specific cleavage is usually found in the virus core in the absence of its precursor [55]. P13-E/L-FL virions contain less P28K than P13 or P13-H5-FL. As we did not analyze the adjacent bands, we cannot say whether or not decrease in the cleaved form of the L4 protein is associated with the presence of the P28K precursor in purified virions. There are reduced levels of early RNA and protein production in cells infected by L4R deficient vaccinia virus particles [56], which could also explain decreased levels of mRNA throughout the course of macrophage infection by P13-E/L-FL virus (data not shown). P13-E/L-FL virions yielded additional bands containing host proteins. Tubulin β chain has been found previously in non-recombinant vaccinia virus IMV where it can form up to 0.7% of protein content [57]. We found an increased amount of tubulin β chain in the core fraction together with increased FL in P13-E/L-FL. MALDI analysis of the core fraction yielded GAPDH. This enzyme, which is involved in many different cellular processes, has also been found incorporated in human immunodeficiency virus type 1 (HIV-1) virions [58]. GAPDH binds actin filaments *in vitro *[59] and therefore could be incorporated in the complex with actin. Actin has been identified in virions of VACV [57] several other DNA viruses and HIV.

## Conclusions

The expression of FL highly influenced the behavior of recombinant vaccinia virus in dependence on the level of its expression. The production of FL driven by the strong synthetic promoter resulted in decreased growth of P13-E/L-FL in confluent macrophage cell line as well as in its limited multiplication *in vivo *when inoculated in mice. The specificity of the effect of high FL levels on virus multiplication was confirmed using the reversion mutant. The inhibition of *in vivo *replication was mirrored in the level of antibodies against vaccinia virus. A strong FL expression in CV1 and HeLa cells changed not only the host range of the recombinant but also the basic protein contents of virions. The major proteins - H3L and D8L - which are responsible for the virus binding to the cells, and 28 K protein that serves as a virulence factor were changed in the membrane portion of P13-E/L-FL viral particles. There also were changes in the core portion as a consequence of poorer protein processing (multiple larger, uncleaved proteins in comparison with the control virus) and the increased content of cellular proteins. The overexpression of FL also resulted in its incorporation into the viral core of P13-E/L-FL IMV particles. In contrary to the equimolar ratio of the glycosylated to the non-glycosylated form of FL in transfected cells, the recombinant virus incorporated mainly the smaller, non-glycosylated FL. We have shown that the overexpression of the FL gene in VACV results in attenuation of the virus *in vivo*.

## Materials and methods

### Plasmids

The pHUFLT3L plasmid containing the coding sequence of the soluble isoform of FL (ID number - U29874) was obtained from Immunex (now the part of Amgen Inc.). The plasmids pSC59-H5-FL and pSC59-E/L-FL have been described earlier [33]. The expression plasmid pBSC-FL was prepared by ligation of an EcoRI fragment of pHUFLT3L plasmid carrying the FL coding sequence with the pBSC plasmid [60] cleaved with the same enzyme. The pTK^+ ^plasmid was derived from pGS20 [61] by excision of the EcoRI fragment. The pD357 plasmid [62] containing an E.coli β-galactosidase gene under the control of the P7.5 promoter was used for replacement of the C23L and B29R genes by the β-galactosidase gene

### Viruses

Vaccinia virus strain Praha, clone 13 [63] was used as the parental virus. Single recombinants P13-H5-FL and P13-E/L-FL were prepared by the insertion of FL gene into thymidine kinase using plasmids pSC59-H5-FL and pSC59-E/L-FL, respectively, followed by selection in medium supplemented with bromodeoxyuridine. The double recombinants P13-βgal-H5-FL and P13-βgal-E/L-FL were prepared by the insertion of β-galactosidase into the FL-expressing single recombinants using pD357 plasmid and by the selection of the virus forming stable blue plaques after three purification steps. The revertant virus P13-ΔE/L-FL was prepared using the pTK^+ ^plasmid carrying the functional thymidine kinase gene; the selection occurred in 143B cells grown in HAT supplemented E-MEM medium. Virus P13-E7 carrying the E7 early protein of HPV16 has been described earlier [64]. Virus P13-βgal-pS2S was derived from P13-pS2S [65] using pD357 plasmid. Viruses were grown in BSC40 cells, purified by sucrose-gradient centrifugation [66] and titrated in CV-1 cells. The number of virus particles was determined from the optical density measured at 260 nm using the formula 1 U of OD_260 nm _= 1.2 × 10^10 ^viral particles/ml [67]. The ratio was comparable for all the viruses used in the experiments.

### Cell lines

CV-1 and BSC-40 African green monkey kidney cell lines were grown in Modified E-MEM medium (EPL, SEVAPHARMA, Prague) containing bovine serum growth-active proteins but no complete serum [68]. Human embryonic kidney 293T cells [69]kindly provided by J.A. Kleinschmidt, DKFZ, Heidelberg, Germany, human HeLa cell line and J774.G8 mouse macrophage cell line were grown in DMEM (PAA Laboratories, Linz, Austria) supplemented with 10% fetal bovine serum (FBS; PAA Laboratories). The cell lines MOLM9 and 32D were grown in RPMI-1640 medium (Sigma, Saint Louis, MO) supplemented with 10% FBS. The 143B cell line was maintained in E-MEM medium (SEVAPHARMA, Prague) supplemented with 10% FBS. Each medium contained 2 mM L-glutamine, 100 U/ml penicillin, and 100 μg/ml streptomycin.

### Mice

Six-week-old C57Bl/6 (H-2^b^) female mice were obtained from Charles River, Germany. Animals were maintained under standard conditions at the National Institute of Public Health (Prague). The experiments were performed in compliance with Acts Nos. 246/92 and 77/2004 on animal protection against cruelty and Decree No. 311/97 of the Ministry of Health of the Czech Republic, on the care and use of experimental animals. Mice were injected intraperitoneally (i.p.) with 0.5 ml PBS containing sonicated suspension of sucrose-purified particles of rVACV. Mice were anesthetized with halothane (Narcotane, Léciva, Praha) and carotid blood was collected at indicated time intervals.

### ELISA

FL was quantified with an Flt3 ligand ELISA detection kit (R&D Systems GmBH, Wiesbaden-Nordenstadt, Germany) using the capture mouse monoclonal antibody MAB608 (100 ng/well), biotinylated detection goat polyclonal antibody BAF308 (7.5 ng/well), streptavidin-HPR (1:250) or avidin-HPR (1:1000) complex, both obtained from Pharmingen (BD Biosciences, Erembodegem, Belgium), and TMB substrate solution for visualization of the reaction. Samples were measured by an ELISA reader at 450 nm. Standard Flt3 ligand protein (PeproTech EC Ltd, London, UK) was diluted to 500-7,5 pg/ml. Detection of VACV-specific antibodies has been described earlier [70].

### SDS-PAGE and western blot

Infected cells or purified viral particles were extracted with denaturing, reducing sample buffer [71]. Samples were separated by SDS-PAGE in 10% or 12% gels. Proteins were blotted onto a nitrocellulose membrane (Hybond-C Extra, Amersham) and after blocking with 10% skimmed dry milk in PBS, the membrane was incubated with primary antibody BAF308 (anti-FL, R&D Systems GmBH, Wiesbaden-Nordenstadt, Germany) diluted 1:500 or convalescent mouse serum (anti-VACV) diluted 1:50 - 1:100. After washing, the membrane was incubated with rabbit anti-mouse IgG horseradish-peroxidase-conjugated secondary antibody (Sigma-Aldrich, Steinheim, Germany). Proteins were visualized with the ECL Plus system (Amersham).

### Mass spectrometry and protein identification

Electrophoretic gels were stained with Coomassie blue. Selected spots on the preparative gels were excised and destained using 50% acetonitrile in 25 mM ammonium bicarbonate, dehydrated with 200 μl of acetonitrile for 5 min at 30°C and then vacuum-dried (SpeedVac, Thermo Scientific, Waltham, Ma). Gel pieces were rehydrated and proteins were digested for 8 hours at 37°C with 30 ng/μl trypsin (Trypsin Gold Mass Spectrometry Grade, Promega, Madison, WI) in 25 mM ammonium bicarbonate. After digestion, peptides were extracted from gel pieces using step by step extraction with an acetonitrile gradient (15%-60% acetonitrile with 1% trifluoroacetic acid) using sonicator (Elma, Singen, Germany) cooled with ice cubes. Extracted peptides were concentrated in SpeedVac. MALDI mass spectrometry (MALDI/MS) peptide mass fingerprint analysis was used to characterize the digests. The MALDI/MS was performed in a Refelex IV MALDI-TOF mass spectrometer (Bruker). Data were processed by proteomic software Mascot.

### Beta-galactosidase assay

Beta-galactosidase activity was determined according to Miller [72]. The samples of infected cells were frozen and thawed and then centrifuged to remove cellular debris. Beta-galactosidase activity of cell extracts was measured by a colorimetric assay using o-nitrophenyl β-D-galactopyranoside (ONPG). The absorbance of samples was determined at 450 nm.

### Virus neutralization

Sonicated, sucrose purified virus particles were incubated in a minimal volume of PBS, with 0.5 μg of rabbit polyclonal antibody against FL (MBL, Woburn, MA) or with rabbit and mouse anti-VACV serum or negative serum. After a 1-hour-incubation at 37°C, the viruses were used for the infection of confluent cell layers or for the preparation of electron-microscopy samples.

### Electron microscopy

Metal grids were freshly coated with a Formvar (polyvinylformal, Serva) membrane. Five to ten μl of viral suspension (sonicated or antibody-treated) were absorbed to the grid for 10 min and then washed twice with water and twice with 1% phosphowolframic acid, pH 9.0, each time for 1 min. The samples were observed by transmission electron microscope JEM1011 (JEOL, Tokyo, Japan) at indicated magnifications.

### Fluorescent microscopy

The cell monolayer grown on a round glass plate was infected with virus at a MOI of 2 at 37°C for 30 minutes. The inoculum was then removed and cells were cultivated in DMEM. At time intervals not longer than 12 hours post infection (h.p.i.), the cells were washed with cold PBS, fixed for 10 minutes in 4% paraformaldehyde (PA) and permeabilized for 10 minutes in 2% PA with 1% Triton-X100. The remaining PA was neutralized by incubation of samples with 0.1 M glycine in PBS for 10 minutes. After blocking in 10% skimmed dry milk in PBS for 30 minutes, samples were incubated with anti-FL MAB608 (diluted 1:500) and with anti-GM130 or with anti-calreticulin (both diluted 1:100; Santa Cruz Biotechnology) in 5% dry milk in PBS for 30 minutes, washed five times (PBS, 0.2% Tween 20) and incubated with secondary antibody against mouse IgG labeled with Alexa fluor 488 and/or with antibody against rabbit IgG labeled with Alexa fluor 546 (Invitrogen, USA), diluted 1:500 in 5% dry milk in PBS for 30 minutes and counterstained with 1 μg/ml propidium iodide or DAPI (Sigma-Aldrich Gmbh, Munich, Germany). The washed plates were observed by Nikon E600 fluorescence microscope for green, red and blue signals at a magnification of 1000×.

### Fractionation of viral proteins

Virion samples containing equal amounts of proteins (determined by the Bradford protein assay, BioRad) were incubated in 50 mM Tris-buffer with 10 mM MgCl_2_, pH 8.5, at 37°C for 30 minutes and subsequently supplemented with detergents for solubilization of two membrane and two core fractions (soluble lipid envelope fraction "M1", protein-matrix-like "M2", soluble core "C1" and DNA-core fraction "C2") [47]. As the supplements for the membrane and core fractions, 1% NP-40 or 1% NP-40 plus 50 mM DTT and 0.5%DOC plus 0.1% SDS or Laemmli buffer were used, respectively. Each fraction was separated from insoluble proteins by centrifugation at 13000 g for 10 min.

### Quantitative PCR (Q-PCR)

Mice were anaesthetized with halothane (Narcotan, Léciva, Praha) and sacrificed. The ovaries were dissected, washed in PBS and homogenized. DNA was extracted using DNeasy Tissue Kit (Qiagen). Real-time quantitative PCR was performed as described previously [33].

## Competing interests

The authors declare that they have no competing interests.

## Authors' contributions

KZ participated in the design of the study, performed most of the experiments and drafted the manuscript. PH prepared the succrose purified virus. JK carried out the quantitative PCR. LK performed ELISA for detection of anti-vaccinia antibodies and provided valuable background for manipulation with vaccinia virus, cell cultures and mice. MS carried out MALDI. SN conceived of the study, participated in its design and coordination and helped in elaboration of the manuscript. All authors read and approved the final manuscript.
